# Associations between adherence, depressive symptoms and health-related quality of life in young adults with cystic fibrosis

**DOI:** 10.1186/s40064-016-2862-5

**Published:** 2016-07-29

**Authors:** K. B. Knudsen, T. Pressler, L. H. Mortensen, M. Jarden, M. Skov, A. L. Quittner, T. Katzenstein, K. A. Boisen

**Affiliations:** 1 The Department of Infectious Diseases, Copenhagen University Hospital, Rigshospitalet, Blegdamsvej 9, 2100 Copenhagen, Denmark; 2 Cystic Fibrosis Center Copenhagen, Department of Pediatric and Adolescent Medicine, Copenhagen University Hospital, Rigshospitalet, Copenhagen, Denmark; 3 The Department of Public Health, University of Copenhagen, Copenhagen, Denmark; 4 The University Hospital Center for Health Research (UCSF), Copenhagen University Hospital, Rigshospitalet, Copenhagen, Denmark; 5 Department of Psychology, University of Miami, Coral Gables, FL USA; 6 Center of Adolescent Medicine, Department of Pediatric and Adolescent Medicine, Copenhagen University Hospital, Rigshospitalet, Copenhagen, Denmark

**Keywords:** Cystic fibrosis, Adherence, Depression, Quality of life, Mental health, Young adults

## Abstract

**Background:**

Cystic fibrosis (CF) is a life shortening disease, however prognosis has improved and the adult population is growing. Most adults with cystic fibrosis live independent lives and balance the demands of work and family life with a significant treatment burden. The aim of this study was to examine the relationships among treatment adherence, symptoms of depression and health-related quality of life (HRQoL) in a population of young adults with CF.

**Methods:**

We administered three standardized questionnaires to 67 patients with CF aged 18–30 years; Morisky Medication Adherence Scale, Major Depression Inventory, and Cystic Fibrosis Questionnaire-Revised.

**Results:**

There was a response rate of 77 % and a majority of the young adults (84 %) were employed or in an education program. Most participants (74 %) reported low adherence to medications. One third (32.8 %) of the participants reported symptoms of depression. HRQoL scores were especially low on Vitality and Treatment Burden, and symptoms of depression were associated with low HRQoL scores (p < 0.01) with medium to large deficits across on all HRQoL domains (Cohen’s d 0.60–1.72) except for the domain treatment burden. High depression symptom scores were associated with low adherence (r = −0.412, p < 0.001).

**Conclusions:**

Despite improved physical health, many patients with CF report poor adherence, as well as impaired mental wellbeing and HRQoL. Thus, more attention to mental health issues is needed.

## Background

Cystic fibrosis (CF) is a genetic, chronic and fatal disease that is often diagnosed early in life. CF is a multi-organ disease which primarily affects the pancreas, digestive and pulmonary systems, leading to risk of diabetes, malnutrition and chronic pulmonary infections (Spoonhower and Davis 2016). From the time of diagnosis until death, the treatment regimen is a substantial part of daily life. This regimen includes chest physiotherapy, inhalation treatments, oral and intravenous medications. Some of the treatments are daily and preventive, while others are therapeutic and may be increased during pulmonary exacerbations (O’Sullivan and Freedman 2009). Optimization of medical treatment has improved survival, however respiratory failure is the main cause of death. Thirty years ago, few individuals with CF reached adulthood. Today, the average life expectancy for newborns with CF is approximately 40 years and is projected to be more than 50 years if mortality continues to decrease at the rate observed between 2000 and 2010 (MacKenzie et al. 2014; Ratjen et al. 2015). As a result, there is an increased focus on adolescents’ transition into adulthood, as well as issues of adherence, mental health and health-related quality of life (HRQoL) (Quittner et al. 2014a, b; Sawicki et al. 2011). In qualitative studies, young adults have described their lives with CF as a complex balance between leading a healthy normal life and feeling different from peers due to the burdensome aspects of disease management (Jessup and Parkinson 2010; Badlan 2006). Many find it challenging to integrate the prescribed treatments into their busy lives, and this has been found to affect psychological well-being and HRQoL (Sawicki and Goss 2015).

Recently, studies have found poor adherence (Quittner et al. 2014a) and a high prevalence of depression in CF populations (Quittner et al. 2014b; Quittner et al. 2008; Modi et al. 2011). However, both rates of adherence and depression vary between different patient populations and few studies have included the Danish CF population. There is currently no gold standard for measuring adherence to prescribed medications, and rates of adherence also vary considerably depending on how adherence is measured (Modi et al. 2006). The International Depression/Anxiety Epidemiological Study of CF patients (TIDES), conducted at 154 CF centers in nine countries in Europe and USA, reported elevated rates of depression for adults across countries, according to age, screening measure, and country (Quittner et al. 2014b). Overall, 10 % of adolescents and 19 % of adults had elevated depression scores, depending on the screening tool. The Hospital Anxiety and Depression scale (HADS) yielded the lowest scores (11 % elevated) versus 27 % elevated on the Center for Epidemiological Studies Depression Scale (CES-D) (Quittner et al. 2014b). Note however, that HADS has been criticized both for a lack of sensitivity and a failure to represent the major symptoms underlying depression diagnosed using the DSM-IV or ICD-10 (Cameron et al. 2008). Denmark was not included in TIDES study and thus, the prevalence of depression among Danish adolescents and adults with CF is not known. It was hypothesized that adults with CF who had symptoms of depression would have lower rates of adherence and impaired HRQoL. The aim of this study was to examine rates of adherence to prescribed treatments, assess symptoms of depression, evaluate HRQoL, and test the associations between adherence, depression and HRQoL in young adults with CF.

## Methods

### Material and participants

This was a cross-sectional study using well-validated questionnaires administered to young adults aged 18–30 years with a confirmed diagnosis of cystic fibrosis (CF). Participants were recruited from November 2013 to December 2014 at a specialized, outpatient clinic for CF at the Copenhagen University Hospital, Rigshospitalet, which is one of the two CF centers in Denmark. The Danish CF population consists of 460 people and about 300 are followed at Rigshospitalet. Participants were recruited during their regular outpatient visit, were informed about the study and gave their oral informed consent to participate by completing the questionnaires. For patients who did not attend the outpatient clinic regularly, questionnaires were sent by mail with a self-addressed, stamped return envelope. The questionnaires were provided with an explanatory letter indicating that a final consent was inferred from completing and returning the questionnaire.

Data on demographic and clinical characteristics were extracted from the Danish CF Registry for the entire CF population at Rigshospitalet aged 18–30 years, and are described as the mean of all measurements obtained in 2014. Results from lung function tests were obtained via chart review, measured by forced expiratory volume in one second (FEV_1_). FEV_1_ values in liters were transformed into the percent of predicted (FEV_1_ %) as described by Hankinson et al. (1999).

### Patient-reported outcome measures

Three questionnaires were included and filled out at one time point: Morisky Medication Adherence Scale (MMAS-8) (Morisky et al. 2008) is a validated self-report questionnaire that measures adherence to prescribed medications, includes specific medication-taking behaviors, and identifies common barriers to poor adherence. It is an 8 item test, 7 items rated dichotomously as “yes/no” responses (0 or 1 point), and one item rated on a 5-point frequency scale from 0 (never or rarely), 0.25 (once in a while), 0.50 (sometimes), 0.75 (usually) to 1 (always) point. A total score on the MMAS ranges from 0 to 8 points; <6 point indicates low adherence, 6 < 8 points indicates medium adherence and 8 points reflects high adherence (Morisky et al. 2008). MMAS-8 has been translated to several languages and many psychometric tests of the translated instrument have been performed. The scale has demonstrated good sensitivity but only moderate internal consistency. The scale has also demonstrated convergent validity with electronic measures and is a reliable tool to determine medication adherence (Morisky et al. 2008; Arnet et al. 2015).

The Major Depression Inventory (MDI) (Bech et al. 2001) is a self-administered instrument that measures depressive symptoms. It consists of 10 items that assess the ICD-10 and DSM-IV (WHO 1993; APA 1994) symptoms of major depressive illness. On a 6-point Likert scale, individual items measure how much of the time these symptoms have been present during the past 24 h. The scale ranges from 0 (symptoms have not been present at all) to 5 (symptoms have been present all of the time). To fulfill the ICD-10 criteria of moderate to severe depression, at least two of the three core symptoms need to be present. The three core symptoms are: depressed mood, lack of interest, and lack of energy. Scores range from 0 to 50; categorized into ‘No depression’ (0–19 points), ‘Mild depression’ (20–24 points), ‘Moderate depression’ (25–29 points), ‘Severe depression’ (more than 29 points) (Bech et al. 2001). Sensitivity and specificity for DSM-IV has been found to be high for the MDI (Olsen et al. 2003). A recent psychometric evaluation of the MDI as a depression severity scale confirmed these cut-off scores (Bech et al. 2015). Participants screened positive for moderate-severe depression, with a MDI score >24, were contacted and offered a consultation with a psychologist.

The Cystic Fibrosis Questionnaire-Revised-Teen/Adult version (CFQ-R) (Quittner et al. 2012; Bregnballe et al. 2008) is a well-validated, disease-specific HRQoL instrument for individuals with CF ages 14 years and older. It has undergone extensive reliability and validity testing and is now considered the “gold standard” measure of HRQoL for CF. The CFQ-R assesses demographic information (e.g., age, education) and measures twelve domains of functioning: Physical Functioning, Vitality, Emotional Functioning, Eating Disturbances, Treatment Burden, Health Perceptions, Social Functioning, Body Image, Role Functioning, Weight, Respiratory Symptoms and Digestive Symptoms. Scores are standardized and range from 0 to 100, with higher scores indicating better quality of life (Quittner et al. 2012).

### Statistical analysis

Means and standard deviations were calculated for MMAS-8, MDI and CFQ-R, as well as demographic and clinical characteristics. Independent t-tests were used to determine if there was a difference between participants and non-participants. To test how the CFQ-R was associated with other characteristics, a one-way multivariate analysis of variance (MANOVA) was used to test overall differences.

MANOVAs were carried out to test whether independent variables, such as gender, age group (18–23 years/24–30 years), marital status (single/partner), level of education (lower education/high school/college or university), work/education ability versus work/education disability, adherence (MMAS score <6/MMAS score ≥6) and depression (MDI score <20/MDI score ≥20) were associated with the twelve domains of CFQ-R. To control for increasing Type I error rate, statistically significant MANOVAs were followed up by univariate ANOVAs to test domain-specific differences. To estimate the magnitude of the association between symptoms of depression, ability to work and HRQoL, effect sizes were calculated by subtracting the means and dividing the result by the pooled standard deviation, resulting in Cohen’s d. Given the variable and small sample sizes, calculation of the pooled standard deviation was adjusted with weight for sample sizes according to Hedges and Olkin (Lakens 2013).

To determine the strength of the linear relationship between MMAS-8 and MDI, we calculated a Pearson correlation coefficient. Logistic regression was performed to ascertain the effects of gender, age, civil status, level of education, work/education ability related to health on adherence (MMAS score <6/MMAS score ≥6) and depression (MDI score <20/MDI score ≥20), respectively. Data management and analysis were performed using IBM SPSS Statistics version 20. A p value <0.05 was considered statistically significant.

## Results

During the study period 90 patients aged 18–30 years were registered at the Copenhagen CF Center with a confirmed CF diagnosis. Three patients lived abroad during the study period and were excluded. Among the 87 eligible patients, 67 completed the questionnaires (response rate 77 %). These measures were completed at the outpatient clinic by 61 patients; however 12 patients who did not attend the clinic regularly received the questionnaires by mail, and six of these patients returned the completed questionnaires. In total, 20 patients did not fill in the questionnaires, four refused to participate and 16 did not respond to inquiries. No significant differences were found between the participants versus non-participants on any demographic characteristics (see Table 1). Most participants (84 %) were employed or in an education program and 3 % were unemployed. Nine participants (13 %) were unable to work or study due to health problems.

**Table 1:** Demographic and clinical characteristics of participants

	Study participants	Non-participants	*p* value
Number	67	20	
Age *mean (range)*	24.1 (18–30)	22.6 (18–30)	0.09
Females, *n* (%)	38 (59)	8 (40)	0.20
BMI (kg m^2)^, *mean* (SD)	21.8 (3.6)	21.2 (2.2)	0.40
FEV1 % predicted*, mean* (SD)	72.2 (23)	74 (22)	0.80
FEV1 (≤40 %), *n* (%)	7 (11)	1 (5)	
FEV1 (41–70 %), *n* (%)	25 (38)	7 (35)	
FEV1 (>71 %), *n* (%)	33 (51)	12 (60)	0.70
Chronic pulmonary infection^a^, *n* (%)	40 (62)	10 (50)	0.30
CFRD, *n* (%)	18 (28)	7 (35)	0.40
Single^b^, *n* (%)	33 (49)	NA	
Lower education, *n* (%)	21 (31)	NA	
High school, *n* (%)	18 (27)	NA	
College/university, *n* (%)	19 (28)	NA	
Employed or studying, *n* (%)	56 (84)	NA	
Incapacitated to work/study, *n* (%)	9 (13)	NA	

*CFRD* cystic fibrosis related diabetes

^a^Pseudomonas aeruginosa, Achromobacter xylosoxidans and Burkholderia species

^b^Single status is self-reported and defined as not having a romantic partner

### Adherence

MMAS-8 was completed by 66 participants. Forty-nine (74.2 %) scored in the low adherence range (MMAS score <6), 12 (18.2 %) had medium adherence (MMAS score 6 < 8), and five (7.6 %) reported high adherence (MMAS score = 8) (Table 2).

**Table 2:** Distribution of MDI and MMAS scores

	N (%)
No depression (MDI score 0–19)	45 (67.2)
Mild depression (MDI score 20–24)	9 (13.4)
Moderate depression (MDI score 25–29)	5 (7.5)
Severe depression (MDI score 30–50)	8 (11.9)
Low adherence (MMAS score <6)	49 (74.2)
Medium adherence (MMAS score = 6 < 8)	12 (18.2)
High adherence (MMAS score = 8)	5 (7.6)

### Depression

The MDI was completed by all participants. MDI scores above 20 indicated symptoms of depression and were endorsed by 32.8 % of the participants; 19.4 % expressed symptoms of moderate or severe depression (Table 2); of these, 77 % had consulted a psychologist or psychiatrist and/or received antidepressant medication. The three remaining participants were contacted and offered a consultation with a psychologist, but all claimed to be feeling well, and had been sad or worried the day they completed the questionnaires. Females were more likely to report more symptoms of depression on the MDI; with a female/male OR of 5.1 (95 % CI 1.03–25.3) for moderate/severe depression scores.

### Quality of life

The CFQ-R scores across the 12 domains of functioning are displayed in Table 3. Vitality and Treatment Burden were the lowest scores (47.1–52.9), indicating poor HRQoL; in Body Image and Eating Disturbances were the highest scores (77.8–83.4).

**Table 3:** CFQ-R domain scores in relation to depression scores and employability

	Total (N = 67)	No depression (N = 45)	Symptoms of depression (N = 22)	No depression versus depression symptoms		Work/education ability (N = 58)	Work/education disability (N = 9)	Work/education ability versus disability	
	Mean (SD)	Mean (SD)	Mean (SD)	Effect size^a^ d	p value^b^	Mean (SD)	Mean (SD)	Effect size^a^ d	p value^b^
Physical functioning	72.8 (27.3)	80.7 (22.6)	56.5 (28.9)	−0.98	*0.001*	79.3 (21.8)	31.0 (22.0)	−2.21	*0.001*
Role limitations	73.5 (26.0)	81.7 (21.2)	52.6 (27.0)	−1.25	<*0.001*	80.6 (18.2)	22.6 (12.4)	−3.09	*0.001*
Vitality	47.1 (25.3)	58.1 (21.2)	26.4 (15.5)	−1.62	<*0.001*	50.2 (24.4)	27.2 (22.3)	−0.93	*0.01*
Emotional functioning	69.3 (20.8)	78.4 (13.8)	50.0 (21.1)	−1.72	<*0.001*	73.0 (18.7)	45.2 (17.6)	−1.50	*0.001*
Social functioning	70.8 (20.7)	77.0 (19.2)	57.7 (14.4)	−1.10	<*0.001*	73.2 (19.7)	55.4 (21.2)	−0.90	*0.02*
Body image	77.8 (23.3)	84.8 (17.1)	61.1 (27.8)	−1.12	<*0.001*	81.3 (20.7)	55.6 (27.8)	−1.18	*0.002*
Eating disturbances	83.4 (25.2)	89.4 (20.6)	70.4 (31.7)	−0.77	*0.007*	85.6 (24.1)	69.1 (28.7)	−0.67	0.07
Treatment burden	52.9 (21.8)	56.1 (21.7)	47.5 (22.5)	−0.39	0.17	54.0 (21.2)	45.7 (25.7)	−0.38	0.3
Health perception	55.2 (29.1)	67.7 (23.7)	29.0 (22.9)	−1.65	<*0.001*	60.0 (27.6)	24.7 (19.1)	−1.21	*0.001*
Weight problems	72.2 (38.6)	78.3 (34.8)	55.6 (42.8)	−0.60	*0.04*	79.5 (32.6)	25.9 (43.4)	−1.68	*0.001*
Respiratory symptoms	62.9 (25.0)	69.5 (21.5)	50.0 (26.5)	−0.84	*0.008*	65.8 (23.7)	45.1 (27.0)	−0.76	*0.02*
Digestive symptoms	74.0 (24.4)	80.1 (21.2)	62.3 (27.0)	−0.77	*0.03*	73.8 (25.1)	75.3 (20.6)	0.06	0.9

^a^Cohen’s d corrected effect sizes are classified as small (d = 0.2), medium (d = 0.5), large (d = 0.8) and very large (d = 1.3) ^b^Values in italic represent p < 0.05

### Associations between adherence, depression and quality of life

To test how the CFQ-R was associated with independent variables, such as gender, age, civil status, level of education, work/education ability, adherence and depression, a one-way multivariate analysis of variance (MANOVA) was used to test overall differences. There were statistically significant difference between CFQ-R and depressive symptoms (p < 0.01) and work/education ability (p < 0.001). Thus, one way ANOVA analyses of the main effect of depressive symptoms and work/education ability were performed and result of the effects on the twelve domains are shown in Table 3. Participants who reported depression and work/education disability scored significantly lower on all domains of the CFQ-R, except Treatment Burden. We found medium to large effects for these associations. We found significantly higher HRQoL scores for those who had a MDI score <19 (‘No depression’) compared to those who had a MDI score 20 < 24 (‘Mild depression’), with similar results for Physical Functioning (p = 0.048), Role Limitations (p = 0.009), Vitality (p = 0.003), Emotional functioning (p = 0.001), Social Functioning (p = 0.02), Health Perception (p = 0.001) and Respiratory Symptoms (p = 0.03). No associations were found between the MMAS-8 scores and the CFQ-R.

**Fig. 1:**
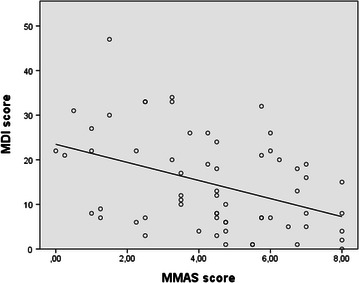
Correlation between depression scores and adherence scores

Depression and adherence scores were negatively associated, increases in MDI scores were associated with decreases in MMAS scores (see Fig. 1), indicating that symptoms of depression were associated with worse adherence, r = −0.412, p < 0.001. Pearson correlations were performed with and without the outlier, with no difference in the results.

An increase in MDI scores was moderately correlated with a decrease in MMAS scores, *r* = −0.412, *p* < *0.001.*

## Discussion

In this cross sectional study, which examined rates of adherence and depression in relation to HRQoL, we found a large number of young adults with CF who reported poor adherence, high rates of depression and impaired HRQoL.

The MMAS-8 was a useful screening tool, identifying a majority of participants with low adherence (74 %). In a large national US study, both pediatric and adult patients with CF picked up 50 % or fewer prescribed medications (Quittner et al. 2014a). A recent systematic review found generally poor adherence in patients with CF, with a lack of consensus on how to measure adherence (O’Donohoe and Fullen 2014). Generally higher rates of adherence have been found in studies using self-report measures in comparison to more objective measures (e.g., medication possession ratios, electronic measures) (Modi et al. 2006). The MMAS-8 is short (8 questions), easy to understand, and adaptable for different kinds of medications (Culig and Leppee 2014). According to the WHO, non-adherence among patients with chronic diseases is about 50 % in developed countries (Sabaté 2003). Several studies have suggested that barriers to CF treatment adherence include lack of time, forgetting, social stigma, and treatment burden (George et al. 2010; Bregnballe et al. 2011). Treatment complexity in CF has increased over the past decades and high treatment complexity scores have been inversely related to Treatment Burden on the CFQ-R, indicating that increasing complexity is associated with worse perceptions of burden (Sawicki et al. 2013). In a qualitative study, patients reported that it was a challenge to fit these time-consuming treatments into daily life, and some patients were intentionally non-adherent to permit spontaneity, independence and social activities (George et al. 2010).

In the current study, we found low scores on the CFQ-R Treatment Burden domain, indicating high perceptions of treatment burden across patients, and this effect was independent of work ability or symptoms of depression. We found a moderate negative correlation between depression and adherence to treatment. Fidika et al. (2014) reported a decline in lung function (FEV1 % predicted) in adolescents and adults with CF with elevated symptoms of depression in a 2-year observation study. Our results support their hypothesis that depressed patients may fail to perform their prescribed treatments due to mental health problems, such as depression. Thus, our findings suggest that treating depression is an important target for intervention, and may have positive effects on lung function and other health outcomes. In addition, high perceptions of treatment burden and poor adherence should be addressed using systematic, behavioral interventions. Brief, behavioral interventions have been shown to be most effective in individuals with chronic conditions (e.g., HIV) (Gross et al. 2013). These interventions should be tested in the Danish CF population.

In our study, one third of the participants screened positive for depression and 19.4 % were in the moderate to severe range. These Danish results are similar to the highest rates reported in TIDES study, with rates of 29 % reported at centers using the CES-D as a screening tool (Quittner et al. 2014b). In our study, we used the MDI, a screening tool used to analyze the prevalence of depression in the general population (Olsen et al. 2003). Good sensitivity and specificity, as well as responsiveness to antidepressant treatments, have been reported for the MDI (Bech et al. 2001; Konstantinidis et al. 2011). The MDI was not developed for patients with CF and there is a risk of identifying false positives. However, it is worth noting that the group of participants who had symptoms of mild depression, compared to those with no symptoms of depression, scored significantly lower on HRQoL domains that measure psychosocial functioning. Thus, even if the group of patients with mild depression symptoms is overestimated, it could be an indication of a need for more psychosocial support. These findings are in agreement with the consensus statements from The International Committee on Mental Health in Cystic Fibrosis which recommended that adolescents and adults with CF whose depression or anxiety is in the mild range should be provided with information about depression and anxiety and receive preventive or supportive psychological interventions (Quittner et al. 2016). All clinically elevated scores from screening tools should be followed up with a more in-depth clinical interview. Importantly, 77 % of the 13 participants who reported moderate or severe symptoms of depression had already consulted a psychologist or psychiatrist and/or received antidepressant medication, suggesting that using the MDI as a screener provided valid data. Unfortunately, we have no information on the length or outcomes of these psychological or pharmacological treatments. Given that their depressive symptoms have persisted, it may be important to evaluate evidence-based psychological interventions, tailored specifically to the needs of those with CF (Quittner et al. 2016). Current evidence for psychological interventions is insufficient, but cognitive behavioral therapy have shown the most promising results according to a recent Cochrane review by Goldbeck et al. 2014.

Comparing the CFQ-R results of this study with the national, normative data from the US Epidemiological Study of CF (ESCF) which had 4679 participants (ages ≥14 years) (Quittner et al. 2012), our scores on all domains, except Physical Functioning, Body Image and Weight were lower for this cohort of young Danish adults. The scores in this study are comparable to those reported in a former Danish CF study of patients with moderately reduced lung function (FEV_1_ 41–70 % predicted) (Bregnballe et al. 2008), however more than 51 % of the participants in our study had a lung function value greater than 70 % of predicted. This study confirms the results of prior studies which have found that depression is related to worse HRQoL on the CFQ-R. (Havermans et al. 2008; Riekert et al. 2007; Yohannes et al. 2012).

Young adults are likely still transitioning to adult roles, including starting a career and family, which according to Chick and Meleis (1986) may be characterized by disorientation, distress, irritability, anxiety and depression. In our study, we found markedly reduced HRQoL among participants who were not studying or working due to health problems. A link between worse lung function and reduced HRQoL scores among individuals with CF was described by Riekert et al. (2007), however, lung function alone does not reflect an accurate picture of a patient’s working capabilities (Frangolias et al. 2003), and in a previous study, decreased work capacity was correlated with HRQoL, but not with lung function (Targett et al. 2014). Having a job clearly has positive effects, such as improved financial prosperity, social participation, self-esteem and psychological well-being (Targett et al. 2014). Thus, a combination of inability to work and increasing health problems negatively impact HRQoL.

This study includes the majority of young adults with CF in Copenhagen, with a high response rate. The Copenhagen CF population is generally in a state of good health, with relatively high lung function and BMI, despite severe CF-mutations (Knudsen et al. 2015). Treatment of Danish patients with CF has been centralized since 1962, and these patients are followed at the CF center every month and monitored closely. The limitations of this study include the relatively small sample size and the use of questionnaires that have not been validated in a CF population. The Danish translation of MMAS-8 was conducted with a great deal of rigor, but it has not been validated in a psychometric study. MMAS-8 measures ‘total’ adherence, thus differences in adherence between types of treatment cannot be assessed. The MDI is validated and widely used in Denmark in the general population; however it is not a common screening tool for depression worldwide. The International Consensus Guidelines on Mental Health in CF recommended using the PHQ-9 and GAD-7 to screen patients with CF each year, beginning at age 12 (Quittner et al. 2016). According to Danish law, questionnaire studies can be done without the participants’ written consent; however, this implies that individual health data cannot be obtained. Consequently, it was not possible to investigate how FEV_1_ correlated with work disability and HRQOL.

## Conclusions

Our study demonstrated that among young adults with CF, adherence to prescribed treatments is low, symptoms of depression are prevalent and quality of life is impaired. Our study confirms that symptoms of depression have a significant and negative effect on HRQOL. Finally, we demonstrated that the MMAS-8 is a useful tool for measuring adherence among individuals with CF. This study supports the recommendations for annual screening of depression and anxiety in those with CF ages 12 years and older and highlights the mental health needs of young adults with CF that need increased attention.

## Abbreviations

CF: cystic fibrosis
HRQoL: health-related quality of life
TIDES: The International Depression/Anxiety Epidemiological Study of CF patients
HADS: The Hospital Anxiety and Depression scale
CES-D: Center for Epidemiological Studies Depression Scale
FEV_1_: forced expiratory volume in one second
MMAS-8: Morisky Medication Adherence Scale
MDI: The Major Depression Inventory
CFQ-R: The Cystic Fibrosis Questionnaire-Revised-Teen/Adult version
CFRD: cystic fibrosis related diabetes
