# Erratum to: Associations between adherence, depressive symptoms and health-related quality of life in young adults with cystic fibrosis

**DOI:** 10.1186/s40064-017-3789-1

**Published:** 2017-05-31

**Authors:** K. B. Knudsen, T. Pressler, L. H. Mortensen, M. Jarden, M. Skov, A. L. Quittner, T. Katzenstein, K. A. Boisen

**Affiliations:** 1 The Department of Infectious Diseases, Copenhagen University Hospital, Rigshospitalet, Blegdamsvej 9, 2100 Copenhagen, Denmark; 2 Cystic Fibrosis Center Copenhagen, Department of Pediatric and Adolescent Medicine, Copenhagen University Hospital, Rigshospitalet, Copenhagen, Denmark; 3 The Department of Public Health, University of Copenhagen, Copenhagen, Denmark; 4 The University Hospital Center for Health Research (UCSF), Copenhagen University Hospital, Rigshospitalet, Copenhagen, Denmark; 5 Department of Psychology, University of Miami, Coral Gables, FL USA; 6 Center of Adolescent Medicine, Department of Pediatric and Adolescent Medicine, Copenhagen University Hospital, Rigshospitalet, Copenhagen, Denmark

## Erratum to: SpringerPlus (2016) 5:1216 DOI 10.1186/s40064-016-2862-5

After publication of the original article (Knudsen et al. 2016), the following corrections, and addition of citations were requested:

On page 2, Method section: subsection: Patient-reported outcome measures:

It is an 8 item test, 7 items rated dichotomously as “yes/no” responses, and one item rated on a 5-point frequency scale. A total score on the MMAS ranges from 0 to 8 points; <6 point indicates low adherence; 6 < 8 points indicates medium adherence; and 8 points reflects high adherence (Morisky et al. 2008). MMAS-8 has been translated to several languages, and many psychometric tests of the translated instrument have been performed. The scale has demonstrated good sensitivity but only moderate internal consistency. The scale has also demonstrated convergent validity with electronic measures and is a reliable tool to determine medication adherence (Morisky et al. 2008, 2011; Arnet et al. 2015; Krousel-Wood et al. 2009).

On page 4, Table 2 and on page 5, Fig. 1, the copyright statement “Use of the ©MMAS is protected by US and International copyright laws. Permission for use is required. A license agreement is available from: Donald E. Morisky, MMAS Research (MORISKY) 16636 159th Place SE, Renton WA 98058, dmorisky@gmail.com” must be included as a footnote.

On page 7, References, the following references should be included:

Krousel-Wood MA, Islam T, Webber LS, Re RS, Morisky DE, Munther P (2009) New medication adherence scale versus pharmacy fill rates in seniors with hypertension. Am J Manag Care 15(1):59–66

Morisky DE, DiMatteo MR (2011) Improving the measurement of self-reported medication nonadherence: final response. J Clin Epidemi 64:258–263. PMID:21144706
